# The Island of Time: Yélî Dnye, the Language of Rossel Island

**DOI:** 10.3389/fpsyg.2013.00061

**Published:** 2013-02-18

**Authors:** Stephen C. Levinson, Asifa Majid

**Affiliations:** ^1^Max Planck Institute for PsycholinguisticsNijmegen, Netherlands; ^2^Radboud University NijmegenNijmegen, Netherlands

**Keywords:** time, diurnal tenses, space-time mapping, gesture, Yélî Dnye, Papuan languages, linguistic relativity, cross-cultural

## Abstract

This paper describes the linguistic description of time, the accompanying gestural system, and the “mental time lines” found in the speakers of Yélî Dnye, an isolate language spoken offshore from Papua New Guinea. Like many indigenous languages, Yélî Dnye has no fixed anchoring of time and thus no calendrical time. Instead, time in Yélî Dnye linguistic description is primarily anchored to the time of speaking, with six diurnal tenses and special nominals for *n* days from coding time; this is supplemented with special constructions for overlapping events. Consequently there is relatively little cross-over or metaphor from space to time. The gesture system, on the other hand, uses pointing to sun position to indicate time of day and may make use of systematic time lines. Experimental evidence fails to show a single robust axis used for mapping time to space. This suggests that there may not be a strong, universal tendency for systematic space-time mappings.

## Introduction

### Overview

This paper describes the temporal system of a language spoken in unusual geographical and cultural isolation. The basic conception of time, it turns out, is cyclical without calendrical fixed points (e.g., without dates, named days of the week or named months, and without recurring festivals at fixed intervals – except where borrowed recently from English). Time is clearly of some considerable cultural concern: there are six tenses, partings express the expected time lapse till the next meeting, certain events follow another at a fixed interval of days, and there is keen awareness of movements of sun, moon, tide, and seasons (where seasons are vague and determined not calendrically but by shifts in weather, crops, migrating birds or fish, and changes in vegetation).

### Yélî Dnye and its speakers

Yélî Dnye is a Papuan, i.e., non-Austronesian language, with no proven relationship to any other language. It is spoken on an island c. 450 km offshore of Papua New Guinea by around 5000 people, the sole inhabitants of the island (35 km by 10 km in size), for whom it is the primary language. There has been about 60 years of intensive mission activity (now in abeyance), which introduced English as the medium of instruction. The island is served by no regular transport, and consequently there is little market economy and little evidence of state institutions.

The language is highly complex with 90 phonemes (including sounds known to no other language), complex irregular morphology in huge paradigms, and extensive verb suppletion. It is ergative both in morphology and also (very unusually) in syntax. Henderson (1995) and more extensively Levinson (in preparation) provide grammatical descriptions of the language.

## Space and Time Expressions in the Language

### The linguistics of space

A full account of the spatial system of the language is given in Levinson and Wilkins (2006). The main frame of reference is an absolute (or at least geocentric) frame, opposing a mountain-sea axis, and an east-west axis which is aligned with the prevailing winds which dominate the affordances of travel by sea. As you go around the island, the mountain-sea axis will rotate, while the east-west one naturally remains fixed. Cognitively speaking this system is slightly odd: if you ask people to make an array as they saw it on the other side of the island, they will make the array so that the East-West orientation is held constant, but the mountain-sea axis is reversed. Director-matcher tasks with two or more objects in table-top space are invariably solved using this system as the main linguistic way of fixing orientation (using terms that gloss as “up” = East, “down” = West, “the direction of the hills” = inland, “the direction of the sea” = seawards (Levinson, 2006) (p. 183ff). Spatial adverbs and verbs of motion are hooked into the same coordinate system (e.g., *koko* “ascend” = go East, *ghîî* “descend” = go West).

From this absolute orientation system a “force dynamics” model is abstracted, which covertly structures a lot of vocabulary, opposing “with a force” vs. “against a force,” with an orthogonal “across the line of force.” Thus there are specific nouns and verbs for going with, against, or across the directions of wind, river, or uphill ridge. This generalized system is expressed in intransitive and (separately) transitive verbs of motion, verbs of carrying, place names, etc. (see Burenhult and Levinson, 2008; Levinson, 2008; Levinson and Burenhult, 2009).

Yélî Dnye also has a quite rich system of distinctions in the intrinsic (or object-oriented) frame of spatial reference, drawing on body part terms like *kpadama* “back,” *‘nuwo* “nose, point,” on more abstract sidedness terms like *kuwó* “back side,” *kada* “front side,” *wéni* “right side,” *t:anê* “left side,” and on more projective spatial terms like *nuw:o* “facing,” *kêêlî* “between.” There is also a rich topological set of around 15 spatial topological postpositions, and a very through-going set of three positional verbs (“sitting,” “standing,” “hanging”), where the exact same semantic oppositions recur in verbs of putting and taking without any clue from the lexical form (i.e., there are underived verbs meaning “put.sitting,” “take-sitting,” etc.; see Levinson and Brown, 2012).

**Figure 1:**
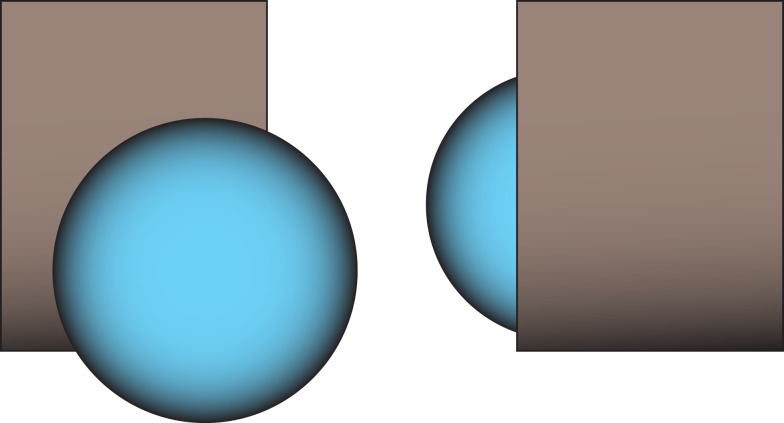
**Ambiguity of “the ball is in front of the block” in Yélî Dnye**. The expression describes either scene.

These intrinsic (object-oriented) terms have possible interpretations in the relative (egocentric) frame of reference, but only in circumstances where the figure object (theme) is being located with respect to an unfeatured (facetless) ground object, as in “the boy is in front of the tree.” There are terms for “left” (*t:anê*) and “right” (*wéni*) but these are normally interpreted intrinsically – “left of Jim” or “left of the dog” is ordinarily interpreted in terms of the referent object’s left/right, and similarly for “in front” and “behind.” Where a relative interpretation is forced, the interpretation of “the ball in front of the block” is ambiguous between the block being between me and the ball and the ball between me and the block (see Figure 1) – both an index of the marginality of the egocentric system, and a causal factor in its lack of use.

There is also a rich deictic system, with demonstratives opposing three degrees of distance (*ala* “this right here,” *kî* “that,” *mu* “that yonder”), evidentiality (*kî* “certain, observed” vs. *wu* “uncertain, unobserved”), and exophoric (all the above) vs. anaphoric reference (*yi*). Deictics are also incorporated into portmanteau verbal inflections, so no lexical “come” vs. “go” opposition is required.

### The linguistics of time

The visitor to Rossel Island quickly realizes time matters on this island without clocks. Greetings vary, as in English, according to the time of day (morning/midday/afternoon/night). More interestingly, partings must specify whether one expects to see the other person one, two, or three or more days from now – provided for this, there are special mono-lexemic ordinal terms for days up to 10 (“see you on the tenth day from now”), and a productive system beyond 10 (see Table 1).

**Table 1:** **Special terms for days from 2 back to 20 ahead**.

Day	Yélî Dnye term	English translation
−2	*m:ii tuwó*	Day before yesterday
−1	*ma*	Yesterday
0	*awedê*	Today
1	*mââ*	Tomorrow
2	*m:ii*	Day after tomorrow
3	*pyêmê*	Day after day after tomorrow, i.e., 3 days from now
4	*p:aamê*	Fourth day
5	*lyimê*	Fifth day
6	*wêêmê*	Sixth day
7	*pyimê*	Seventh day
8	*waamê*	Eighth day
9	*tómê*	Ninth day
10	*yomê*	Tenth day
11	*y:oo mye mââ*	Tenth day plus tomorrow, i.e., 11 days from now
12	*y:oo mye m:ii*	Tenth day plus day after tomorrow, i.e., 12 days from now
13	*y:oo mye pyêmê*	Tenth day plus day after the day after tomorrow…
20	*y:oo mye y:ême*	20 Days from today

Verbal inflections and suppletive verb roots distinguish six tenses, according to the day of the event: earlier today, yesterday, the day before yesterday, or further in the past; later today, tomorrow, the day after tomorrow, or later in the future. Example (1) illustrates sentences in the punctual aspect, in which only four tense distinctions are made:

**Table:** 

(1)	*doo pîpî*	“He was eating it the day before yesterday or before”
	*dê ma*	“He ate it earlier today”
	*Ø ma*	“He ate it yesterday”
	*Ø ndîî*	“He ate it the day before yesterday or before”

Tables 2 and 3 provide an overview of where the full distinctions are made, and what they mean. All these terms are deictically anchored in time with respect to *now*, the moment of speaking. Note that even imperatives are tensed for immediate vs. later action. There are thus extensive devices for marking and counting time in diurnal units from the deictic center, the time of speaking.

**Table 2:** **Tense oppositions in different moods and aspects**.

Tense	Mood					
	Indicative		Habitual		Imperative	
	Cont	Punct	Cont	Punct	Cont	Punct
Future	√ Distal	√ Prox	Ø	Ø	√	√
Immediate future	√ Prox	Ø				
Present	√ Prox		√	√		√
Immediate past	√ Prox	√ Prox			Ø	Ø
Near past	√ Distal	√ Prox				
Remote past	√ Distal	√ Distal	√			

**Table 3:** **The meanings of the tenses and the correlated temporal adverbials labels for tenses come from Henderson (1995)**.

Tenses	Semantic extension	Parallel lexical adverbial
Future distal	Tomorrow or later	*mââ* “tomorrow”
		*m:ii* “day after tomorrow”
Immediate future	Later today	*awêde* “today”
Present	Now	*ala ngwo* “right now”
Immediate past	Earlier today	*awêde* “today”
Near past	Yesterday	*ma* “yesterday”
Remote past	Day before yesterday	*m:iituwó* “day before yesterday”

As mentioned, there is a rich set of deictic pronouns, making three distinctions of distance from the speaker: *ala* “this near me” (in or within grasp), *kî* “that” (unmarked, mid-distance), *mu* “that yonder” (distant), *ye* “that close to you”; in addition *mwada* “far side” can be used as in *mwada mwada dini ghi ngê*, “far.side far.side time part adverbializer” meaning “far in the future.” In combination with time units these can denote near or far units: *ala wiki* “this week,” *mu dini ghi* “that time part” (that period), etc. However, there are very few indigenous time units of this sort – *wiki* is an English loan, *dini ghi* could denote any period from an hour to a century. There are four terms that designate seasons (*nt:eemi*, *m:ââ*, *mbyw:aa*, *kpî*) but these do not exhaust the year but rather indicate periods of the year characterized by winds from certain directions, low tides, etc. The term *d:ââ* for moon can be used to designate a (rough) lunar month; *wo* “light” can be used to designate the diurnal unit, *mgîdî* “dark” can be used to designate night, *m:ââ* “season of low tides” can be used to designate the annual cycle (although this may be modeled on English year). This seems to exhaust the indigenous time units.

The temporal expressions so far described are deictic or used in expressions designating times or events with respect to now, the deictic center. But the language also has an effective system for expressing the temporal relations between events. The language makes much use of two aspects, one punctual, the other continuous, across all tenses and moods. This, together with special temporal constructions (with no spatial meanings) indicating “while” or “as soon as,” etc., allows one to readily encode notions like “While Xing, he Y’d,” “As soon as he X’s, we’ll Y,” “He X’d as he was Y-ing,” etc. Spatial notions like “before” and “after” do not seem to play a central part in time designations – when employed, they inherit all the ambiguities of their spatial counterparts: *kada n:aa kwo* “front I’m going” is idiomatic for “I’m going ahead (of you),” while *a kada dê ghê* “my front he went” would mean “he went ahead of me.” For that reason the ordinal *mwiyé* “first” is likely to be employed.

Most tense adverbials can be introduced by a special temporal postposition *ngê* without spatial meaning, so *Monday ngê* means “on Monday” (all days of the week, months, etc., are recent English loans). Some intrinsically temporal expressions can be introduced bare, without any adposition or adverbializer, as in:

**Table:** 

(2)	*kââdîmââkêlî n:aa*	*m:uum:uu*
	noon period	1s.Fut.Motion see.Cont
	“I’ll see you noonish”	

This is similar to the bare introduction of place names in Yélî Dnye spatial descriptions. Given intrinsic time denoting phrases (e.g., parts of the diurnal cycle, expressions like “tenth day,” etc.) and these means of making time adverbials, there is little need in this language for extensive borrowing of time expressions from the language of space. Areas of overlap are illustrated in Table 4, and mainly consist of a few topological postpositions, just two dimensional adjectives (meaning “tall/long” vs. “short”), a handful of spatial nouns with time uses, and the deictics “this,” “that,” and “yonder.” Bear in mind that this list exhausts the space-time mappings in language.

**Table 4:** **The limited overlap between spatial and temporal descriptors**.

	Yélî Dnye expression	English translation
Topological postpositions	*2 o’clock 3 o’clock kêêlî ghi*	“Between 2 and 3 o’clock” (English calque)
	*July k:oo*	“In(side) July”
	*April u kuwó March*	“(Lined-up) behind April is March”
	*Easter chono*	“Easter is close”
	*Mgîdî ‘nuknî ‘nuknî (p:uu)*	“(Attached to) the intestines/inside of the night”
Dimensional adjectives	*dye ghi daadîî*	“A long/tall time”
	*dye ghi dêêkwédi nê t:ââ*	“I waited a short time”
Spatial nominals	*têdê*	“Place or time of an event”
	*mwandiyé u kêténi*	“Morning its direction,” “mid-morning”
	*u kuwó*, e.g., *u kuwó myaa t:aa*	“Its behind; after it in time,” e.g., “he arrived later”
	*u kada*, e.g., *kada n:aa kwo*	“Its front; before it,” e.g., “I’m standing (going) ahead”
Deictics	*ala*, e.g., *ala wiki*	“This,” proximal deictic, e.g., “this week”
	*kî*, e.g., *kî wiki*	“That,” distal/unmarked deictic (evidentially certain), e.g., “that week”
	*mu*, e.g., *mu mééni dé*	“Yonder” far distal deictic, e.g., “those-far months, previous months”

A special remark about the terms “behind, after” and “in front, before” in Table 3. It was noted above that the relative frame of spatial reference is hardly used, and only partially conventionalized, so that “the ball is in front of the cube” would be ambiguous. The same ambiguity recurs in the temporal domain, so one can say either of the following intending the same obvious reading that Tuesday comes immediately before Wednesday:

**Table:** 

(3)	*Tuesday u kuwó Wednesday*
	Tuesday it’s behind Wednesday
	“Tuesday (is) behind Wednesday, i.e., precedes”
(4)	*Tuesday u kada Wednesday*
	Tuesday its front side Wednesday
	“Tuesday (is) before Wednesday”

Although this, and further examples in Table 3, may seem to be clear space to time mappings, there is reason to doubt this in many of the cases. The prototype use of *kada/kuwó* is for spatial *events*, namely going first in line or last in line. These of course have both a temporal and spatial interpretation – space/time is fused. Likewise *mwandiyé u* *kêténi* (“morning it’s direction, i.e., mid-morning”) presumably refers to the sun’s position, a space-time fusion. The remainder of the overlapping terms seem to rely on introduced calendrical notions, and are probably calques based on English.

Many temporal adverbials are complex expressions, and these typically employ the words *dye* *ghi* “time part,” as in *dye ghi yintómu* “time part all, i.e., always,” or *u kuwó dini ghi n:ii ngê* “its back time part that.one time.adverbializer, i.e., After that….” The nominal *ghi* means “part, piece,” implying a particulate model of time. It is not clear that there is any equivalent of the English metaphors of time passing us by, coming or going (the first author has heard *Christmas ka pwiyé knî*, “Christmas Cont.Pres3 + ProxDeictic go, i.e., Christmas is coming,” but we believe this kind of locution is based on English in a mission context). More natural, anyway, is to speak in terms of time and us moving together, as in *m:ââ kami p:uu a nmî kaa dmi*, literally “year new attached we are accompanying it,” i.e., “We are accompanying the new year (it’s coming soon).” For making appointments or setting dates (in terms of days from now), one can talk about “bringing” a feast “closer” or “taking” it “further” into the future, utilizing the space-time fusion of “bringing/taking” events.

Compared to English, these are few and marginal overlaps in the description of space and time; instead, the two domains are treated linguistically as basically separate except where they are naturally fused in events, casting some doubt on the universal naturalness of space/time mapping (see also Sinha et al., 2011, 2012).

## Spatial Representations of Time

There are no indigenous material representations of time. These are a people without pictorial conventions or elaborate visual art beyond basketry. A few people on the island are likely to have imported calendars (much in demand for help in the setting up and staggering of the many feasts and ceremonies), and school children will be taught English calendrical notions. Most Rossel Islanders are literate to at least some degree in English (and some read every scrap of newspaper that makes its way to the island); just a few can read Yélî Dnye as printed in the SIL New Testament translation – the orthography employs many diacritics and multigraphs due to the 90 phonemes, and people find this hard to read. Practical literacy mostly involves keeping lists, e.g., of shell money debts.

**Figure 2:**
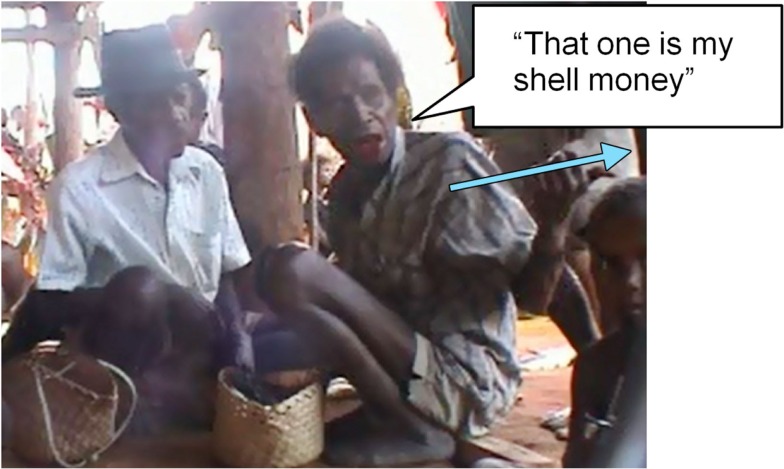
**Pointing in the veridical direction to indicate a person’s identity**.

But the main representation of time other than spoken language is gesture. To understand this, it is essential to understand the spatial uses of gesture. As mentioned, the major frame of spatial reference is absolute (or geocentric). As a result, when speakers mention a place, a person, a motion event of any kind, they are likely to gesture, and gesture in the “correct” direction. For example, if I’m asking you whether you are going back home, I’m likely to point in the actual direction of your home from the current place of speaking. Figure 2 shows a man pointing awkwardly behind him while saying (we gloss) “That one (pointing to distant home base of girl) is my shell money,” meaning that the indicated girl’s bride price should come to the speaker. In this way a deictic can do the job of referring to a distant particular individual (see Levinson, 2007, for many further examples).

**Figure 3:**
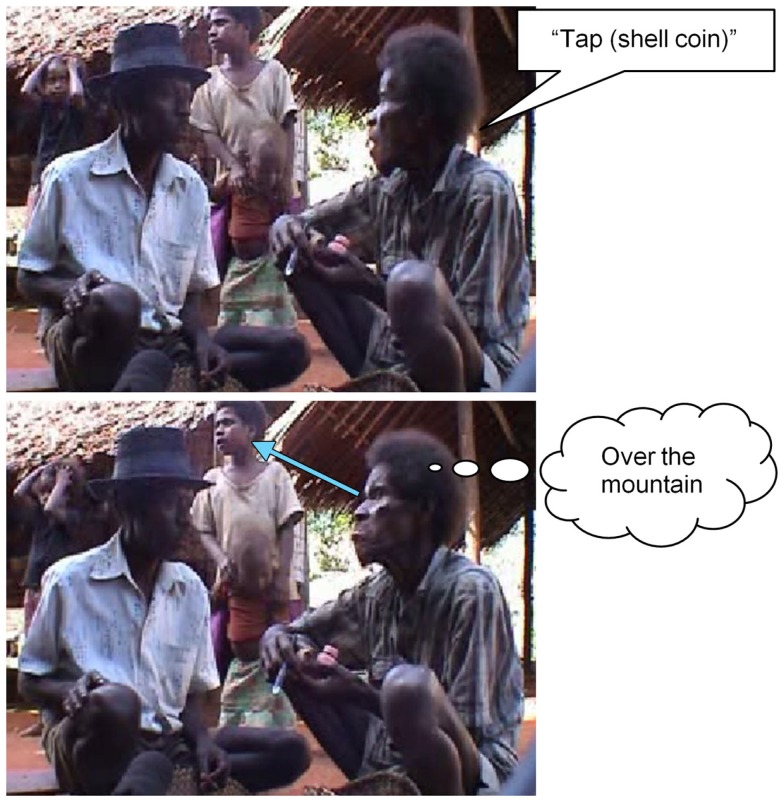
**Eye-pointing spatial location**.

Pointing can also be done with head and eyes, as in Figure 3, where the speaker mentions a very valuable shell coin and wordlessly predicates “it’s up over the mountain there” by producing a gaze-point, combined with a lip-point.

A spatial gesture system of this kind means that gestures are always inspected for directional veracity – you can’t do iconic gestures or diagrammatic hand waving without the danger of being misunderstood (see Levinson, 2003). Consequently, temporal gestures are also constrained. Those most obviously identifiable refer to the movement of the sun or moon, and they point veridically to the past or future location of the celestial body as a way of indicating a specific time of the day or night. These gestures are literally spatial of course, and derive their temporal interpretation from the spatial movement of heavenly bodies.

**Figure 4:**
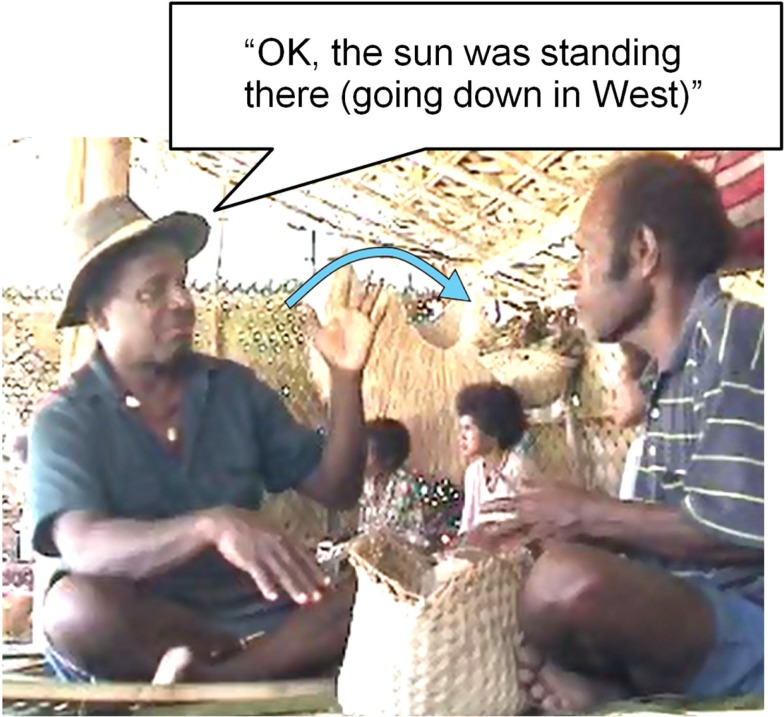
**Time pointing: hand indicates sun position**.

**Figure 5:**
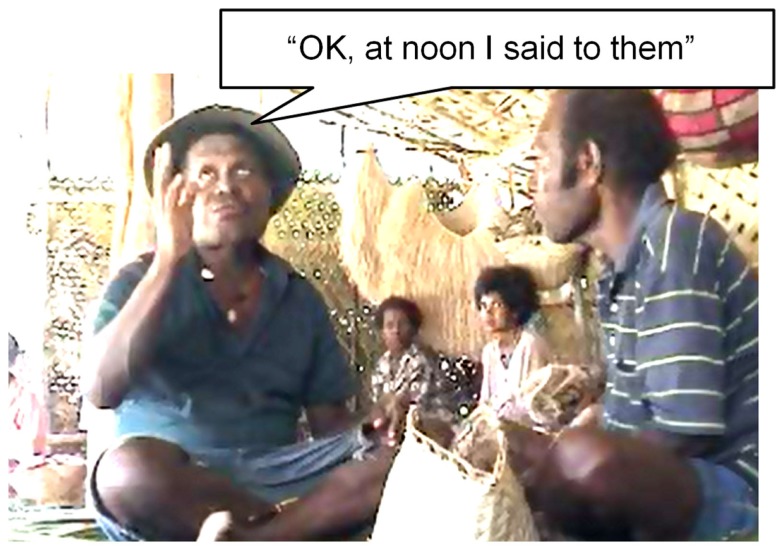
**Time pointing with the eyes and hand, indicating the location of the sun**.

Figure 4 shows a flat hand used to represent the dying sun, veridically represented as going down in the West, while Figure 5 shows that eye-points can be involved in time reference just as in spatial reference (here, combined with a hand gesture, representing vertical position of the tropical sun at high noon). These gestures are used to indicate the time at which events occurred in the past (and are equally used to indicate future times); their interpretation relies on shifting the deictic center to the place and time of the narrated event – indicating that the sun was in such-and-such position when we were there.

**Figure 6:**
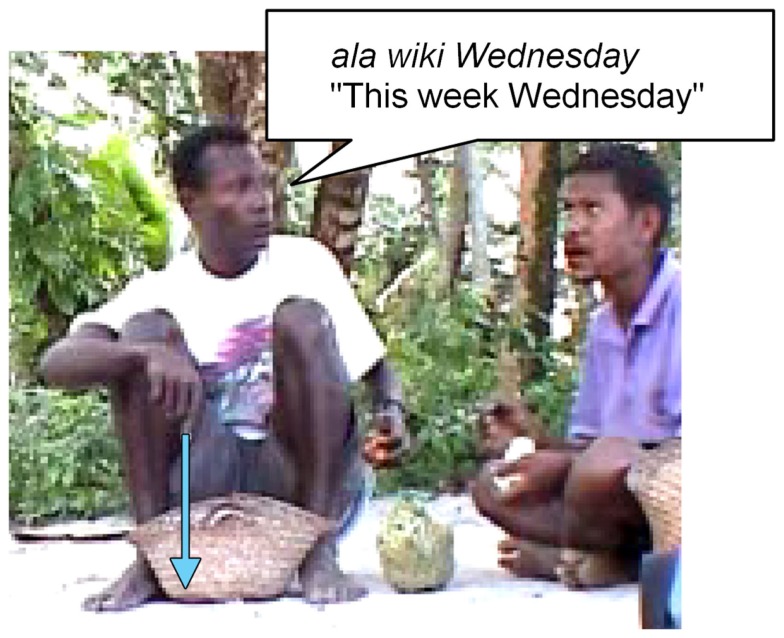
**“Now” = “here**.**”**

Inspection of videotaped conversation suggests that there might be a more abstract representation of time in gesture. First, the deictic *ala* “close to me,” when used in time reference, “this week Wednesday” is sometimes accompanied by a downward gesture as in Figure 6, indicating “now” = “here,” i.e., that there is a unified time-space deictic center.

**Figure 7:**
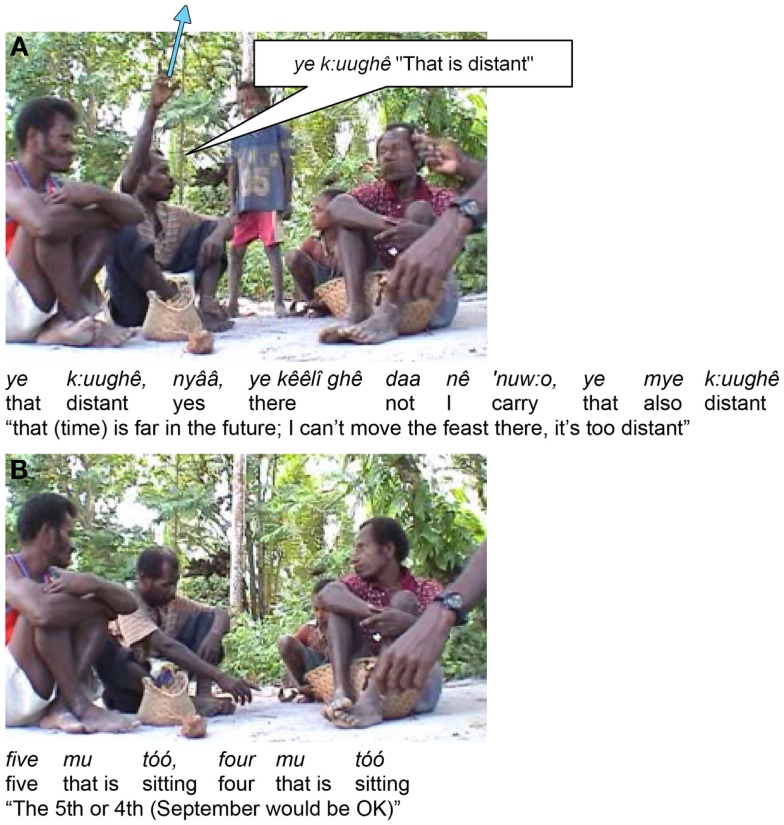
**Time line from high (distant future) depicted in (A), to low (near future) in (B)**.

Second, sometimes in gesture there does seem to be a clear time line. For example, in Figure 7, there is clearly a vertical time line with distant time high, just as spatial distance tends (universally) to be indicated by vertical height.

It appears that horizontal time lines are also used in gesture. The East-West time line has been observed in conjunction with verbs of “bringing up” applied to dates, but this verb also has absolute uses in the spatial domain (it means bring things up East). In general, it is hard to be sure of the consistency of gestural time lines in natural conversation where the affordances of direction of sitting, the possible invocation of spatial motion, and so forth may be involved. Only experimental evidence will resolve the underlying cognitive representations.

## Temporal Reasoning Experiments

In order to explore further the representation of time by Yélî Dnye speakers, two experiments were conducted following Boroditsky et al. (2008) (Boroditsky and Gaby, 2010). In the first experiment participants had to indicate the spatial layout of successive events, e.g., days of the week. In a second experiment, participants were asked to arrange cards that depicted temporal sequences.

To assess whether Yélî Dnye speakers have a conception of the relation of space to time that is distinctly different from the “Standard Average European” one, we also ran these experiments with native speakers of Dutch. Like English, Dutch has rampant space-time correspondences, although there are, of course, myriad subtle differences in the spatial and temporal linguistic encoding devices in these two closely related languages (see, for example, Brée et al., 1990; Van Staden et al., 2006). Critically, however, previous research shows that Dutch speakers – like English, French, German, and Spanish speakers – conceptualize temporal relations along a horizontal spatial axis (e.g., Gevers et al., 2003; see Boroditsky et al., 2011). We, thus, compared Yélî Dnye speakers to a control group of Dutch speakers to test how they spatialized time under equivalent conditions.

### Method

#### Participants

Due to stormy weather and difficulties with river crossings, only 10 native Yélî Dnye speakers took part in the experiments, and the tasks were conducted indoors. Ages varied from approximately 19–50 years; half were male and half female. Four participants had experience in literate tasks off the island (secondary school, primary school teacher’s training, or bible translation), and the sample is in that respect not entirely representative. Participants completed both experiments. An equal number of Dutch participants were matched to Yélî Dnye participants for gender and age *t*(18) = 0.50, *p* = 0.62. It was not possible to match samples for literacy. Only literate or partly literate Yélî Dnye speakers took part in the study, since it was impossible to convey the nature of the task to non-literate speakers. No formal test of literacy is available for speakers, and it was considered culturally inappropriate to ask Yélî Dnye speakers to judge their own literacy skills, therefore one of the experimenters (SCL) estimated literacy for Yélî Dnye speakers on a scale of 0 (not literate) to 10 (high literacy) based on (a) past education, (b) past mission employment, and (c) known use of writing. Dutch speakers were asked to estimate their own literacy *geletterdheid*, which during testing was further explained as “how well can you read and write” on the same 10-point scale. Dutch speakers had higher literacy on average than Yélî Dnye speakers *t*(18) = 3.97, *p* = 0.001.

#### Materials

A compass was used in order to record cardinal direction. A set of standardized coding sheets were used in order to record all responses, including the direction participants were facing, their spatial arrangements, etc.

##### Task 1: Placement of verbal (named) temporal sequences

In the first task, participants were to arrange named temporal sequences. Boroditsky et al. (2008) recommend doing this by asking people to point in space. However, this was too abstract for Yélî Dnye participants, who found the instructions perplexing, and so they were given three pebbles and asked to arrange those for each temporal sequence. All but two of the Dutch participants understood the pointing in space instructions; those who had difficulty understanding instructions was also tested with pebbles.

The English targets for the temporal sequences are given in the table above (Table 5), but it should be borne in mind that the absence of indigenous calendrical notions made it necessary to rely on English loan words or Yélî Dnye expressions which implicated the right contrasts but did not exactly mean them. The exact locutions in both Yélî Dnye and Dutch are given in the appendix.

**Table 5:** **Translation targets for the named temporal sequences in two languages**.

Anchor	First time-point	Second time-point
Today	Yesterday	Tomorrow
Nowadays	Long ago	The future
This week	Last week	Next week
Summer	Spring	Autumn
Midday	Morning	Evening
When you are sleeping	When you are just going to bed	When you wake up from sleeping
Wednesday	Tuesday	Thursday
The age you are now	When you were a baby	When you will be very old
This month	Last month	Next month
This year	Last year	Next year
Noon	Sunrise	Sunset
Middle of the night	Dusk	Dawn

The first six set of oppositions (rows in the table) were given in a fixed order in one block, and the second six at a later point in another block. Participants were facing the opposite direction during the second block.

##### Task 2: Placement of non-verbal temporal sequences

For the second experiment, participants were given a series of picture cards, which depicted temporal sequences (e.g., maturation of an organism, consumption of a fruit, etc.). All materials are available online and the full set was used (see Boroditsky et al., 2008).

#### Procedure

The running conditions were also matched as closely as possible between the two populations: the tasks were conducted indoors, the table for the Dutch testing aligned as it was in the Yélî Dnye setting, the same stimulus materials were used, and facing directions replicated.

Yélî Dnye participants tested on Rossel Island sat in a thatched local house before an imported desk, and were tested one by one by the experimenter with the aid of a native speaker assistant. The long axis of the desk was aligned roughly with the East-West wind axis, with the bush-sea axis perpendicular, so that participants sat facing North, and then later facing South (more precisely the long axis of the table was aligned with c. 110° N). The Dutch participants were tested in the Netherlands indoors with a desk aligned to the same direction as Yélî Dnye speakers.

In the first task, participants were shown three pebbles and instructed in Yélî Dnye as indicated by the following example: *ala chêêpî w:uu pyile tpile knî*, *u mâlo dpî yé té: ‘naa u p:eeni kópu*, *ala chêêpî awêdê*, *ala chêêpî ma*, *ala chêêpî mââ*, “These three pebbles, set them in order. For example (if) this (experimenter places stone in central position) is today, where is yesterday (experimenter holds up another stone), where is tomorrow (experimenter holds up another stone)?” The participant placed the stones on the table however they liked. The participant was asked to rename the identity of the stones. The order of the named stones, and the direction of their alignment in both egocentric and compass directions, was then recorded on coding sheets. The first six scenarios in the table were run through. Then, after an interval (in which the first half of the other task was performed), each participant was tested from the other side of the table, facing in the opposite direction with the remaining six scenarios. Two Dutch speakers failed to fully grasp the original Boroditsky et al. (2008) instructions, and were therefore tested in 2D, as were the Yélî Dnye speakers. The remaining participants conducted the experiment in 3D (with pointing in space). An example of the instructions used: *Dit hier is vandaag. Waar zou je gisteren plaatsen? Waar zou je morgen plaatsen?*, “This over here is today. Where would you place yesterday? Where would you place tomorrow?” The exact temporal expressions used in the two languages are given in the Appendix.

The second task involved aligning four pictures of successive stages of a temporal cycle. In the first block of the task participants were facing one side of the table (South), in the second half they were rotated to the other side of the table facing the opposite direction (North). There were eight sets of sequential cards, half of which were used in each block (counterbalanced blocks; pseudo-random order of sets). Participants were instructed in Yélî Dnye as follows: *ala tpile u mâlo dpî yé té*, *ló n:ii ngmê mwiyé*, *ló n:ii n:ii ngmê u kuwó?* “Put this thing in a line; which comes first, which one is later (behind)?” In Dutch the participants were instructed as follows: *Leg ze in de juiste volgorde*, *zodat je kan zien wat er als eerste gebeurt en wat er later gebeurt*. “Place them [the cards] in the right order so you can, see what happens first and what happens later.”

As described above, participants were tested with Task 1 followed by Task 2 on one side of the table and then rotated to sit at the other side of the table and complete the remaining trials of both tasks. There was an error in recording the cardinal direction for the second sitting for one of the Yélî Dnye participants.

### Results

#### Coding

The data were coded by the experimenters as well as an independent coder. For each trial, coders assigned a dominant orientation to the participants’ response, both in terms of egocentric coordinates (left/right/toward/away) and absolute coordinates (north/south/east/west). Absolute coordinates were determined using the same procedure as Boroditsky and Gaby (2010): the four absolute directions were assigned one of five values (0, 0.25, 0.5, 0.75, or 1) with the value for each trial summing to 1. For example, if the arrangement for a trial was NE then the coding was N = 0.5, E = 0.5, S = 0, W = 0. If it was not possible to determine the linear order of the arrangement then all cardinal values were coded as 0. The average values were then calculated for each participant.

#### Task 1: Placement of verbal temporal sequences

For the Yélî Dnye speakers, there were quite a lot of inconsistent or “incorrect” orders in part attributable to some of the linguistic terms employed – for example the language doesn’t provide clear terms for dawn vs. dusk, and the terms employed may have been obscure; in addition, the absence of indigenous calendrical terms made it hard to come up with a sufficient number of terms to employ.

**Figure 8:**
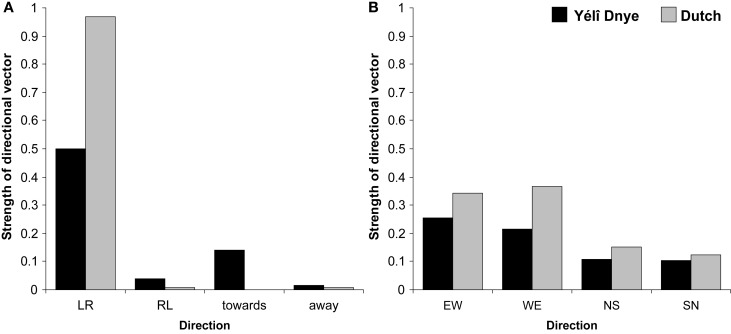
**Placement of verbal temporal sequences**. **(A)** Shows the proportion of left-to-right (LR), right-to-left (RL), toward-the-body, or away-from-the-body responses. **(B)** Shows the proportion of responses across participants that used an east-to-west (EW), west-to-east (WE), north-to-south (NS), or south-to-north (SN) strategy.

Approximately 10% of responses produced across eight different Yélî Dnye participants utilized a non-linear solution. For example, one participant placed “last week” to the left of “this week” but then placed “next week” further to the left of “last week,” so that the final spatial layout was “next week-last week-this week.” Of the remaining linear responses, if there is a dominant pattern, then it is along a left-to-right axis (see Figure 8, which employs the conventions in Boroditsky and Gaby, 2010). (If participants were producing a linear ordering without any preference for a specific layout, then responses ought to be equally distributed across categories at 0.25.) Eight out of 10 participants produced a consistent left-to-right ordering from sitting 1 to sitting 2, although a left-to-right organization was only found for approximately half of the trials, showing Yélî Dnye speakers were not wedded to their use of the left-to-right strategy. The Dutch participants, on the other hand, all produced a linear strategy, and overwhelmingly used a left-to-right spatial layout, as can be seen from Figure 8. One Dutch participant in one trial arranged the cards from right-to-left, and announced whilst doing so that he was trying to be “refreshing.” Yélî Dnye were significantly less likely to use a left-to-right arrangement than Dutch speakers *t*_1_(18) = −5.23, *p* = 0.0001; *t*_2_(11) = −11.41, *p* = 0.0001; *d* = 2.47. In contrast, it appears that Yélî Dnye speakers were more likely to organize temporal sequences toward-the-body *t*_1_(18) = 2.12, *p* = 0.05; *t*_2_(11) = 16.17, *p* = 0.0001; *d* = 0.99.

Yélî Dnye speakers did not, however, demonstrate a preference for an absolute direction in their placement of temporal sequences, as can be seen from Figure 8. If anything, Dutch speakers showed a higher incidence of West-to-East order *t*_1_(18) = 3.47, *p* = 0.003; *t*_2_(11) = 2.26, *p* = 0.05; *d* = 1.64; there was no statistical difference in the East-to-West placements *t*_1_(18) = 1.15, *p* = 0.27; *t*_2_(11) = 0.67, *p* = 0.52; *d* = 0.54. This difference is due to the Dutch consistently using a single (left-to-right axis), while Yélî Dnye speakers did not use a consistent strategy.

We examined the likelihood of producing a left-to-right organization of time in this task as a function of age, gender, and literacy of participants. The only significant association was with literacy *r*(18) = 0.57, *p* = 0.009. This was largely driven by the difference in literacy between the two groups (see also Bergen and Lau, 2012; De Sousa, 2012).

#### Task 2: Placement of non-verbal temporal sequences

**Figure 9:**
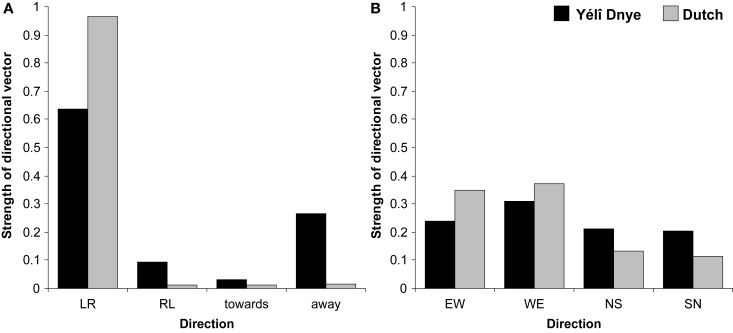
**Placement of non-verbal temporal sequences**. **(A)** Shows the proportion of left-to-right (LR), right-to-left (RL), toward-the-body, or away-from-the-body responses. **(B)** Shows the proportion of responses across participants that used an east-to-west (EW), west-to-east (WE), north-to-south (NS), or south-to-north (SN) strategy.

For the cards task, all participants produced a linear order, suggesting this task was not as hard to understand as the previous verbal sequences task. Five Yélî Dnye participants produced a dominant left-to-right ordering of the cards across the two sittings. Two participants used a different body-based axis, where they placed the cards in order away-from-their-body. Another two participants used a consistent absolute strategy: for one person they ordered the cards in a east-to-west axis, whereas the other person ordered the cards in a dominant west-to-east axis, and this orientation was preserved under rotation across sittings. Dutch speakers overwhelmingly used the left-to-right axis, and this axis was consistent over the two sittings. Figure 9 depicts the dominant strategies collapsing across participants.

As before, Yélî Dnye were significantly less likely to use a left-to-right arrangement than Dutch speakers *t*_1_(18) = −2.56, *p* = 0.02; *t*_2_(7) = −4.69, *p* = 0.002; *d* = 1.21. There was a tendency for Yélî Dnye to organize the temporal cards away-from-the-body *t*_1_(18) = 1.97, *p* = 0.06; *t*_2_(7) = 10.58, *p* = 0.0001; *d* = 0.93. Yélî Dnye speakers did not show more use of an absolute direction. If anything, Dutch speakers appeared to show more east-west arrangements *t*_1_(18) = 2.29, *p* = 0.03; *t*_2_(7) = 3.60, *p* = 0.009; *d* = 1.08. There was no significant difference in the west-to-east arrangements *t*_1_(18) = 0.96, *p* = 0.35; *t*_2_(7) = 2.04, *p* = 0.08; *d* = 0.45. This difference is because of the consistent use of the left-to-right strategy that Dutch speakers applied, in contrast to the more variable responses of the Yélî Dnye speakers.

Once again, we examined the relationship between the likelihood of organizing temporal sequences in a left-to-right fashion against age, gender, and literacy. There was a significant association between left-to-right arrangements and literacy *r*(18) = 0.46, *p* = 0.04. No other association was significant.

#### Individual differences?

The above analyses collapse across individuals, and thus possibly obscure consistent albeit differing individual strategies. Another way to look at the results, therefore, is to calculate the dominant strategy displayed by individuals across sittings. Viewing the results this way suggests that there were quite a few different strategies at play for Yélî Dnye speakers, whereas Dutch speakers all used a dominant left-right organization (see Table 6). Although many Yélî Dnye participants used a left-right coding strategy too, a minority also consistently used an East-West strategy under rotation, a pattern one is very unlikely to encounter in a Western population. A third common strategy was to use a body-centered framework on the sagittal axis (toward/away). Finally, two Yélî Dnye speakers failed to produce a dominant strategy in the pebbles task. Clearly, the results show less consistent spatialization of temporal relations amongst the Yélî Dnye.

**Table 6:** **The dominant strategy by participants in each task**.

Language	Task	EW/WE	NS/SN	LR/RL	Toward/away	No dominant strategy
Yélî Dnye	Verbal sequences	1	0	6	1	2
	Non-verbal sequences	2	0	5	3	0
Dutch	Verbal sequences	0	0	10	0	0
	Non-verbal sequences	0	0	10	0	0

### Discussion

The results from these two experiments suggest that Yélî Dnye speakers have a less conventionalized and less stable mapping of time to space. Whereas all Dutch speakers used a left-to-right organization as the dominant strategy for placing temporal sequences, Yélî Dnye speakers also used a toward-away axis, as well as an east-west axis. This is not to deny that a left-to-right organization was the one attested most often by Yélî Dnye speakers. Our correlational analyses between left-to-right sequencing and literacy certainly conforms with previous findings demonstrating that reading and writing play an important role in how we spatialize temporal sequences (cf. Boroditsky et al., 2011).

Yélî Dnye speakers also differ from the Australian Aboriginal population explored by Boroditsky and Gaby (2010) and Gaby (2012), where participants showed a strong tendency to use an East-to-West timeline. The results support the view that that there simply are no indigenous spatial conventions for representing timelines, witness the individual variation in Table 6. Notable for example is the use of the sagittal axis (toward/away), and, not visible in the pooled results in Figures 8 and 9, the use of consistent absolute timelines by a minority of participants. The tendency to left-to-right order can not be understood in terms of any obvious indigenous systems. The language, as we noted, uses left-right oppositions minimally. It must presumably originate from mission and school, where literacy is important even if reading is a minority enterprise. In the absence of an indigenous convention for temporal spatialization, solving a task that requires a time to space mapping may have directed attention to the only known (and imported) solution. The “school-like” nature of the tasks may also have contributed to the association of the tasks with the left-right bias of literacy. Some evidence that points to the absence of prior convention for time spatialization are the non-linear responses in the pebbles task, and the use of the sagittal rather than transverse axis in the cards task.

Note that given the lack of substantial overlap between time and space in language description, there would be no specific expectation that the predominantly absolute spatial system would be mirrored in the temporal tasks. Even though gesture uses the position of heavenly bodies to indicate time, that use is a literal not a metaphorical use of space (the heavenly bodies really will be there at that time). Nevertheless, there were two consistent (and two inconsistent) users of a fixed absolute direction in the cards task, suggesting that the gestural uses of absolute directions might prime the use of an absolute axis for a novel temporal task.

What is perhaps most interesting is that given this absence of clear mapping of space to time in the language, we find a variety of space-time mappings by Yélî Dnye speakers in tasks designed to explore this.

### Conclusion

Yélî Dnye is a language with a lot of grammatical and lexical resources dedicated to keeping track of time. However, there are almost no indigenous calendrical notions, e.g., named days of the week, named months, fixed beginnings of cycles (years, months, etc.). Instead the linguistic system makes maximal use of times specified in diurnal units from the time of speaking. It also makes extensive use of aspect and special constructions to indicate the relative temporal relations between two events (whose location with respect to *now* will also be coded in tense).

It is clear that in this language most temporal expressions are *not* derived from spatial ones: tense, time adverbs, the main constructions relating two events in time are not derived from the spatial domain. Given the paucity of indigenous temporal units, and a means of calendrically locating them, there is less scope for the use of spatial terms in the temporal domain.

The absolute gesture system also constrains the use of gesture for time, since gestures are regularly inspected for directional veracity. Gestures are demonstrably used to point to movements of the sun and moon to indicate points in the diurnal cycle, and they also seem to be used for abstract time lines, but the evidence from natural discourse remains equivocal as to whether any East-West time line is employed.

The placement tasks for events in series showed use of various time lines, which might be oriented left-to-right, in front and away from ego, or East-to-West. The task imposed a spatial dimension on a temporal representation, and the variability of the results perhaps suggests that this is not a culturally much rehearsed way of thinking.

In conclusion, the main interest of this study is that it casts some doubt on a strong, universal tendency for systematic space-time mappings: these are largely absent from the language, not clearly evident in gesture (except where time is space, as in the movement of celestial bodies), and not coherently reflected across individuals in the temporal tasks. One general hypothesis would be that indigenous languages that lack calendrical notions are also as likely as not to lack systematic space-time mappings: it is only when there is a multiplicity of fixed temporal units that considerations of which “come before” others becomes highly relevant, and the elaborate distinctions from spatial language and thinking are imported into temporal cognition. If so, then the widespread existence of space-time mappings may show more about the cultural elaboration of calendrical notions than about any natural prominence of the parallel between space and time (see Sinha et al., 2011, for independent evidence and speculation along the same lines).

## Conflict of Interest Statement

The authors declare that the research was conducted in the absence of any commercial or financial relationships that could be construed as a potential conflict of interest.

## Appendix

### Temporal sequences in Yélî Dnye

**Table:**

**Anchor**	**First time-point**	**Second time-point**
awêde (today)	ma (yesterday)	mââ (tomorrow)
ala dini ghi (nowadays)	mu dini ghi (past times)	mwada dini ghi (distant time, future implicated)
ala wiki (this week)	m:iituwó wiki (past-days week)	mwada wiki (distant week, future implicated)
m:ââ (low-tide season)	nt:eemî (north-wind season)	mbyw:aa (strong east-wind season)
kââdî mââkêlê (midday)	mw:aandiye (morning)	ntómukwodo (afternoon/evening)
dini ghi ngê nye dpî (when you are sleeping)	mgîtédmyino nye dpuwodpuwo (when you are just going to bed)	yi dini ghi ngê dp:o pyidu (the time when you wake up from sleeping)
Wednesday (English loan from Wednesday)	Tuesday (English loan form Tuesday)	Thursday (English loan form Thursday)
dye ghi n:ii k:oo nye kwo [the time segment that you are now standing (in)]	dini ghi n:ii ngê tpómu nyoo a ya (the time segment when you were a baby)	dini ghi n:ii ngê vy:ee ngê nyoo a ya (the time segment when you will be very old)
ala d:ââmu (this month)	m:iituwo kî d:ââmu ngê (past-days that month)	mwada d:ââmu (far distant month, future implicated)
ala m:eeni (this year)	m:iituwo kî m:eeni ng (past-days that year)	mwada m:eeni ngê (far distant year, future implicated)
kââdî mââkêlê (noon)	kââdî ng:oo (sunrise/half-light)	kââdî u wupwo (sun its going down)
mgîdî ‘nuknî’nuknî p:uu (middle of the night)	kpîmbó/kââdî ng:oo (dawn/sunrise)	kââdî u wupwo (sun its going down)

### Temporal sequences in Dutch

**Table:**

**Anchor**	**First time-point**	**Second time-point**
vandaag (today)	gisteren (yesterday)	morgen (tomorrow)
tegenwoordig (nowadays)	lang geleden (long ago)	de toekomst (the future)
deze week (this week)	vorige week (last week)	volgende week (next week)
zomer (summer)	lente (spring)	herfst (autumn)
middag (midday)	ochtend (morning)	avond (evening)
wanneer je aan het slapen bent (when you are sleeping)	wanneer je net naar bed gaat (when you are just going to bed)	wanneer je wakker wordt van slapen (when you wake up from sleeping)
woensdag (Wednesday)	dinsdag (Tuesday)	donderdag (Thursday)
de leeftijd die je nu hebt (the age you are now)	toen je een baby was (when you were a baby)	wanneer je heel oud bent (when you will be very old)
deze maand (this month)	vorige maand (last month)	volgende maand (next month)
dit jaar (this year)	vorig jaar (last year)	volgend jaar (next year)
‘s middags (noon)	zonsopkomst (sunrise)	zonsondergang (sunset)
midden in de nacht (middle of the night)	schemering (dusk)	dageraad (dawn)
